# Increasing Medical Student Exposure to Neurosurgery: The Educational Value of Special Study Modules, Student Selected Components, and Other Undergraduate Student Projects

**DOI:** 10.3389/fsurg.2022.840523

**Published:** 2022-02-08

**Authors:** Jakov Tiefenbach, Chandrasekaran Kaliaperumal, Andreas K. Demetriades

**Affiliations:** Department of Neurosurgery, Royal Infirmary of Edinburgh, Edinburgh, United Kingdom

**Keywords:** SSM (Special Study Module), SSC (Student Selected Component), undergraduate neurosurgery, medical school curriculum, undergraduate student projects, neurosurgical education

## Abstract

**Objectives:**

Neurosurgery is a tertiary specialty, and exposure to medical students limited. One way to increase engagement and offer experience in neurosurgery is through Student Selected Components (SSC), Special Study Modules (SSM), or independent projects. Our aim was to assess the educational value of such projects and evaluate their effectiveness in exposing students to the field.

**Methods:**

A survey was designed and distributed to Edinburgh University medical students and alumni who completed a neurosurgical project within the last 5 years. The survey was anonymous and collected responses over a fortnight. The results were analyzed in Microsoft Excel 2020 Software.

**Results:**

Twenty-four respondents completed the survey −42% were students and 58% junior doctors. Respondents overwhelmingly enjoyed their project (96%) and reported increased interest in neurosurgery (62%). The project helped improve their knowledge of neurosurgical procedures, pathologies, and/or clinical presentations and allowed connections with the local department. On a Likert scale, 37% felt they gained a good insight into the field. Only 33% felt the project was a good “taster” for the specialty. This is reasonable given that 92% of projects focused on data analysis, and none were designed as clinical attachments. A large number of students had their work published (50%) and presented at conferences (55%).

**Conclusion:**

Lack of exposure to neurosurgery at medical school is a known limitation within a busy curriculum. Selected Components/Special Study Modules/independent projects help students learn about certain aspects of neurosurgery and raise their level of interest. A majority of participants either achieved presentation at conferences or published their work. However, our results suggest neurosurgical projects complement but do not replace traditional clinical attachments in providing insight into the craft of this specialty.

## Introduction

Neurosurgery is a tertiary medical specialty that has been under-represented in the medical school curriculum. The lack of early exposure in recent years has led to a decreased number of residency applications (1). Repeated calls have been made to allocate more time toward neurosurgical teaching at an undergraduate level and to develop a national curriculum (2–4). To our knowledge no universal actions have been taken to address this important issue for neurosurgery or any other similarly sized surgical specialty.

The benefits of increased neurosurgical teaching are clear—medical students could increase their confidence in dealing with neurosurgical emergencies and get clinical exposure to the field. A study by Skarparis et al. found that as many as one-third of final-year medical students in the United Kingdom (UK) have difficulty identifying the need for a neurosurgical referral (5). This is a concerning finding, considering the importance of early presentation and devasting consequences of common neurosurgical emergencies when left untreated. In another study, medical students rated experimental neurosurgical teaching sessions highly and overwhelmingly recognized the benefits of such sessions in their professional and clinical development (6). Taken together, these papers demonstrate there is a clear need and benefits to be gained from increased neurosurgical teaching at an undergraduate level.

Neurosurgical teaching can be achieved in many different formats, including but not limited to bedside teaching, lectures, tutorials, clinics, theater exposure, on-call shadowing, and student projects. The majority of medical schools in the UK do not provide any formal neurosurgical teaching (2); a common route for an interested medical student to get exposure to the field is by completing a neurosurgical project. The scope of these projects can vary greatly, such as conducting an audit, a literature review, a cohort study, or a meta-analysis under the supervision of a local neurosurgical consultant. Students can organize these projects independently or complete them as part of a structured medical school module frequently known as a Student Selected Components (SSC) or Special Study Module (SSM). These projects allow students the opportunity to also extend beyond the scope of the curriculum and either be presented or published nationally or internationally.

The existing literature does suggest that this type of project facilitates student learning and provides an insight into the field. For instance, the Northern Medical School SSC Consortium identified undergraduate student projects as an effective way to develop research skills, acquire knowledge and skills outside the core curriculum, and facilitate students' personal and professional development (7). Furthermore, a paper by Clark et al. argues undergraduate projects can improve career prospects and be an effective method of developing clinical competencies in neurosurgery (8). However, despite all the benefits students can realize through undergraduate projects, one may still argue that they are not the most suitable format for the delivery of undergraduate neurosurgical teaching as they fail to provide sufficient exposure to patients and clinical scenarios for the entire student cohort.

The aim of this study was to assess the educational value of medical student projects in neurosurgery and evaluate their effectiveness in exposing students to the field by getting a preview of what a neurosurgical career means to a medical student. We hope the results may give an insight into both benefits and drawbacks of such projects, as well as offer medical students' perspective on this important topic. We believe this will provide valuable insight for medical schools and relevant national bodies, and encourage them to re-assess the role of undergraduate projects and neurosurgery within the medical school curriculum.

## Methods

An online survey aiming to assess the educational value of student projects in neurosurgery was designed by a senior medical student and a consultant neurosurgeon (JT, AKD). The survey was composed of 19-items, containing Likert scale, multiple-choice questions, and free texts fields. The survey collected information on respondents' demographics, the structure of their project, and their overall impressions. Specifically, respondents were asked to rank the impact the project had on their interest in the field; the level of insight they had gained into the specialty; the amount and type of knowledge they had acquired; the connections they had made with the local department; the research output achieved throughout the project; the impact the project had on their career trajectory and their interest in the field; if they considered the experience to be a good taster for neurosurgery and if it provided a unique type of exposure which could not have been achieved through traditional clinical placements. The full list of questions included in the survey can be found in Table 1.

**Table 1:** The list of questions included in the online survey.

**Question**	**Format**
What is your gender?	Multiple choice
How old are you?	Free text
At what stage of your training are you currently in?	Multiple choice
How much protected time did you have to work on your project?	Multiple choice
When did you complete your project?	Multiple choice
What was the structure of your project?	Multiple choice
What type of project did you do?	Multiple choice
Did the project make you more or less interested in neurosurgery?	Likert scale
Did the project help you gain a better understanding of what a career in neurosurgery might be like?	Likert scale
Did the project help you develop knowledge in one or more neurosurgical procedure, pathology, and/or presentation?	Likert scale
Did you enjoy working on your project?	Likert scale
Would you recommend neurosurgery centered project to your peers?	Likert scale
Do you think that the project was a good “taster” for neurosurgery?	Likert scale
Do you think that the project provided a type of exposure to neurosurgery that could not have been achieved through a traditional clinical placement?	Likert scale
Did the project help you make valuable connections with neurosurgical registrars and consultants?	Likert scale
Have you had work in relation to your project presented at a conference?	Multiple choice
Have you had work in relation to your project published in a medical journal?	Multiple choice
Did the experience help you when applying for a neurosurgical training post?	Multiple choice
Any other comments you would share regarding your neurosurgical project?	Free text

The survey was distributed via e-mail to medical students and junior doctors who had completed a project in neurosurgery over the last 5 years under the supervision of two Edinburgh-based neurosurgical consultants (AKD, CK). The data were exported in an excel sheet and subsequently analyzed via Microsoft Excel 2020 Software.

Participants were informed that the survey was anonymous and were not required to provide any identifiable information. They were also explicitly told the result may be published and offered to withdraw their response at any stage of the project. As an audit of educational experience, no ethical forms were deemed necessary for the completion of this project.

## Results

**Figure 1:**
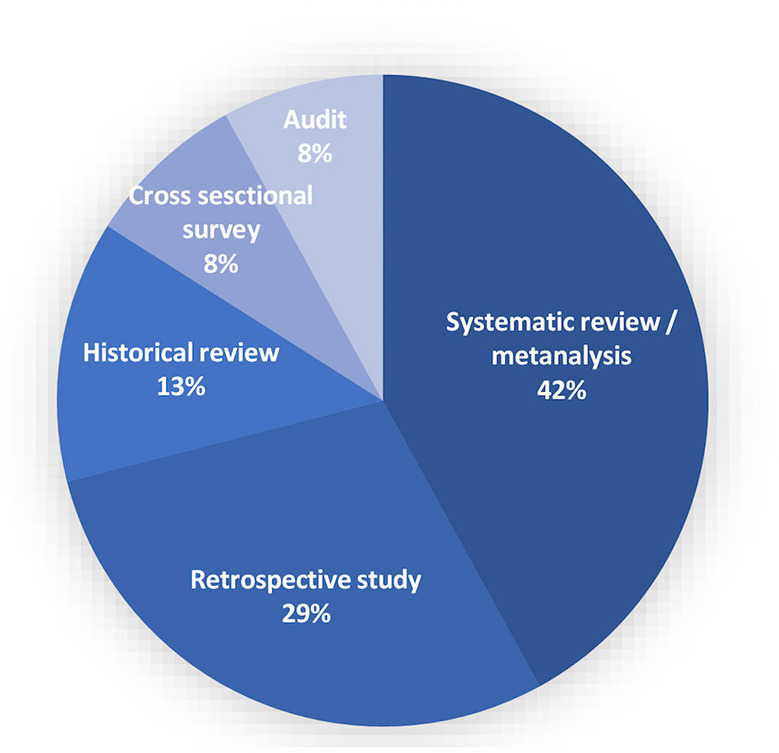
The type of projects undertaken by medical students.

Out of 38 medical students and junior doctors who received the survey, a total of 24 (63%) filled in their responses. The respondent cohort comprised both medical students (42%) and junior doctors (58%) at varying stages of their careers (i.e., foundation programme doctors, core trainees, and pre-specialty clinical fellows). Both male (71%) and female (29%) respondents were represented in this survey; the mean age was 25, with the youngest respondent being 21 and the oldest 30. The majority of projects (58%) were completed as part of an SSC/SSM module, while the remaining 42% were organized independently outside the medical school curriculum. All projects were completed over the last 5 years, with a majority (37.5%) having been completed in 2019/2020. The students had varying amounts of protected time to work on their projects, ranging from no time at all to more than 12 weeks. The type of projects included systematic reviews/metanalyses (42%), retrospective studies (29%), historical reviews (13%), cross-sectional surveys (8%), and audits (8%) (Figure 1).

As many as 62% of respondents reported an increased level of interest in the field after completing their project, while 68% believed the project helped them improve their knowledge in one or more neurosurgical procedures, pathology, and/or presentation. The project benefited students in other ways as well −62% of them published in a medical journal or presented their work at a conference, with another 25% planning to do so in the near future. Students who undertook systematic reviews had the most success in publishing and presenting their work. As many as 79% reported making connections with local neurosurgeons and neurosurgical trainees, which they considered to be of great value in their future careers.

**Figure 2:**
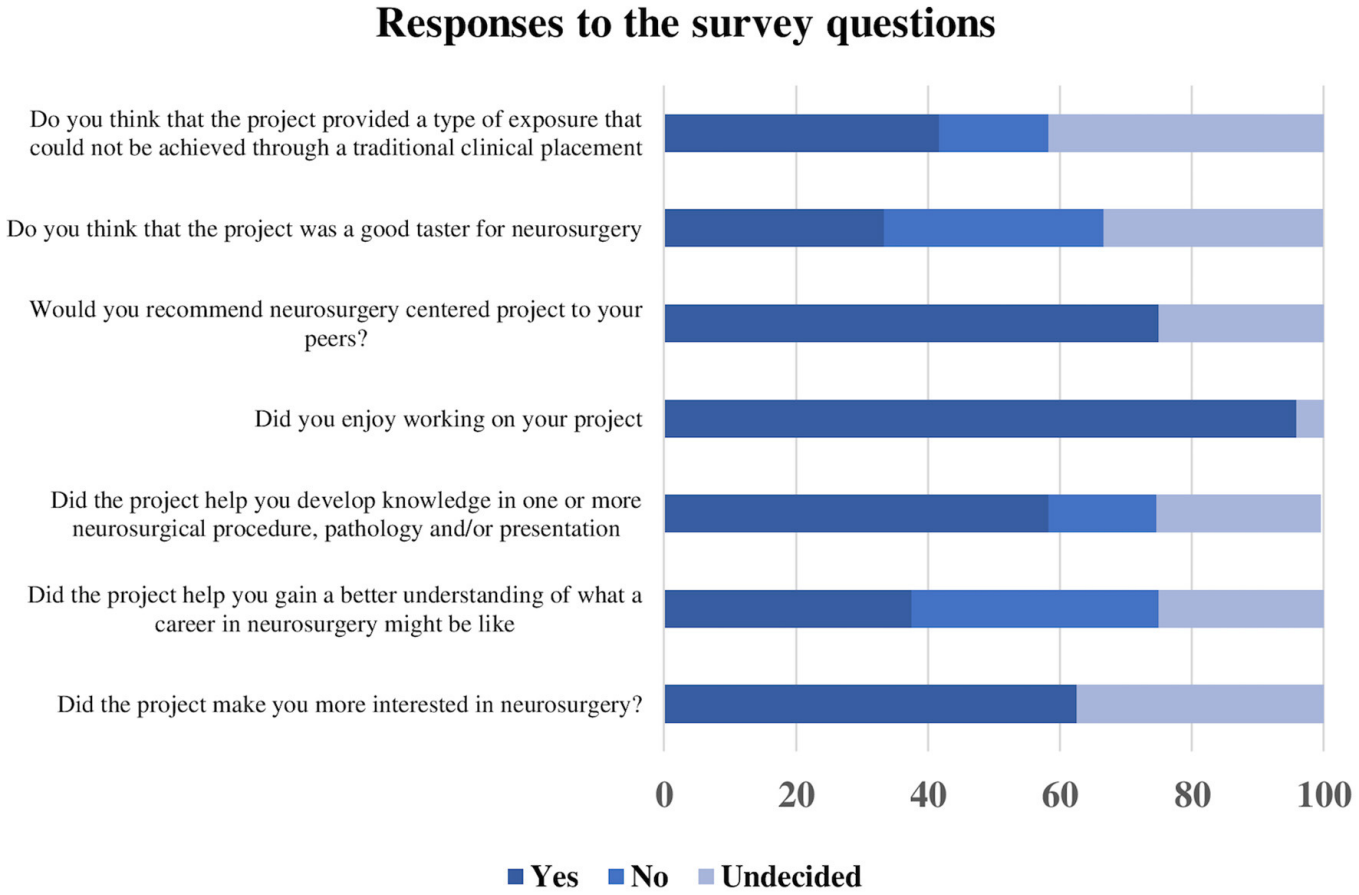
Responses to the survey questions assessing the value of undergraduate projects.

An overwhelming majority enjoyed working on their project (96%) and would recommend a neurosurgical project to their peers (75%). However, only 33% thought this type of exposure to neurosurgery was a good “taster” for this specialty, and only 37% felt the project helped them gain an accurate insight into what a career in neurosurgery would be like (Figure 2). Interestingly, retrospective studies were the type of project that was considered the best “taster” for the specialty, as well as the best way to gain an insight into what a career in neurosurgery might be like.

Finally, the junior doctors who had already applied for a neurosurgical training post did not think their undergraduate project was of much influence in their application. As much as 42% believed that the project provided a type of exposure that could not have been achieved through a traditional clinical placement. This was particularly true for audits, cross-sectional surveys, historical, and literature reviews. The free text field inviting students to leave additional comments did not provide any further insight relevant to this study.

## Discussion

### The Purpose and Benefits of an Undergraduate Project

The purpose of undergraduate student projects has been identified and discussed on multiple occasions. In 2004, the Northern Medical Schools SSC Consortium proposed the purpose of these projects to be: (i) to ensure all students have opportunities to extend their studies beyond the core curriculum, (ii) to develop skills involved in clinical research, (iii) and provide opportunities for personal and professional development (9). The same Consortium revisited the topic in 2005, and produced a list of skills that medical students should be looking to acquire while undertaking undergraduate projects; these included research methods, information gathering, critical analysis and review, data processing, communication skills, self-management, and reflection (7).

The results acquired by our primary survey suggest there are a lot of benefits to these projects, and they do indeed help to develop some of the skills outlined by the Northern Medical School SSC Consortium. Firstly, the projects helped students acquire additional knowledge in neurosurgical procedures, pathologies, and/or clinical presentations which would not have otherwise been covered by their undergraduate curriculum. They have also increased the level of interest in the field and helped them gain more experience in clinical research. A lot of students even published their work in a medical journal or presented at a conference—both of which are very valuable experiences for students' personal and professional development. In addition, projects helped students make connections with local neurosurgical trainees and consultants, which can be incredibly useful for those looking to get further exposure to the field and secure a training post in this competitive specialty.

It is relevant to recognize that the benefits can differ between projects, and largely depend on the assigned supervisor and the type of project the student is attempting to complete. For instance, someone conducting a systematic review will have a very different learning experience from someone running a quality improvement project at the local department. Either way, both students and medical schools should be looking to maximize the value of these projects. A useful starting point to optimize the learning experience of an SSC/SSM comes from a paper published by Riley et al. (10), where 12 highly valuable tips are presented for every participating student to familiarize themselves with.

### The Limitations and Shortcomings of Undergraduate Projects

At the same time, our results suggest there are certain educational aspects where undergraduate student projects did not achieve the maximum desired benefits. For instance, only a third of respondents thought that undergraduate projects were a good taster for the specialty or provided an accurate insight into what a career in this specialty would be like. These results may sound at first discouraging, as one of the aims of undergraduate projects is to allow students additional exposure to their field(s) of interest. However, this is hardly surprising, considering the majority of SSCs/SSMs are designed as research projects focused on primary data. It is worth acknowledging that students seemed keener for a research project rather than a clinical attachment, perhaps due to the competitive pressure to publish or present their work in order to progress in their career.

Another potential drawback of undergraduate projects is that they are self-selecting. Only students with a prior interest in neurosurgery will undertake a project and have an opportunity to learn more about this specialty. As a result, the majority of medical students do not get any exposure to neurosurgery, even though some might have found the specialty stimulating and would have chosen it as their future careers.

### The Future of Undergraduate Neurosurgical Education

Arguably, the best way to ensure every medical student gets an appropriate exposure to neurosurgery would be through a mandatory clinical placement, which is not the case in every medical school (4). Spending a few days or weeks at the local neurosurgical unit, sitting in clinics, scrubbing in theaters, and joining the daily ward rounds would give students an accurate insight into the specialty and help them grasp what a career in this field might be like. It would also ensure all medical students get some kind of exposure to the field, and in such a way adequately supplement student-selected projects. However, the competing pressures of a busy medical school curriculum are well-recognized (7).

In 1993, Dr. Ralph A.W. Lehman, a neurosurgeon at the University of Pennsylvania, posed an important question—will future medical students be taught neurosurgery? (11). Today, most medical schools in the UK do not have any neurosurgical attachments within their undergraduate curriculum. Furthermore, as many as 28% UK medical school representatives do not think neurosurgery should be taught in medical schools at all (2). With the further accumulation of knowledge and increasing constraints on students' time, it is unlikely we will see medical schools universally introducing neurosurgical clinical attachments in the foreseeable future. This has a significant number of downfalls discussed in the previous paragraph, but yet again one needs to be respectful of the constraints faced by medical schools and the relative importance of other specialties in the medical curriculum. Creating medical student-friendly consortium or interest group run by the junior trainees in neurosurgery and with the oversight of neurosurgeons could be beneficial. Such events had already been piloted by Neurology and Neurosurgery Interest Group (NANSIG) and Society of British Neurosurgeons (SBNS), eliciting great interest among the medical students across the country. However, unless a radical shift is made in the way we approach medical education at the undergraduate level, it is likely that interested medical students will have to continue relying on undergraduate projects as their main gateway into this tertiary specialty. Such projects, as shown, are effective in different domains and are currently a complementary aspect to the curriculum.

### Limitations of This Study

While the insights gained from our research are interesting and shed light on this important topic, it is still necessary to address some limitations. Firstly, this was a single center study; thus, our results might not be perfectly generalizable to other universities within and outside the UK. Furthermore, due to the relative size of the specialty, its tertiary level, and limited exposure among medical students, the sample size included only 24 participants. This may have implications for the accuracy and validity of the findings discussed in the paragraphs above. Finally, even though our response rate was satisfactory, this study may have been subjected to non-response bias—it is possible that the students with stronger and more positive feelings toward their project were more inclined to respond and complete the survey.

## Conclusion

Undergraduate student projects, such as SSMs and SSCs, present an excellent learning opportunity for interested students to explore their specialty of interest. This is particularly true if their specialty of interest is neurosurgery, which is peripheral to the modern medical school curriculum due to understandable constraints. However, our primary survey and existing literature suggest these types of projects, while very beneficial in many ways, do not provide sufficient insight into the specialty and are not an adequate replacement for a traditional clinical placement. It is hoped that medical schools and national bodies responsible for the development of undergraduate medical school curricula will consider these findings and their implications in adapting medical student exposure to a tertiary specialty such as neurosurgery.

## Data Availability Statement

The original contributions presented in the study are included in the article/supplementary material, further inquiries can be directed to the corresponding author.

## Author Contributions

AD: contributed to the study concept. JT and AD: contributed to the design. All authors contributed to the data analysis and manuscript.

## Conflict of Interest

The authors declare that the research was conducted in the absence of any commercial or financial relationships that could be construed as a potential conflict of interest.

## Publisher's Note

All claims expressed in this article are solely those of the authors and do not necessarily represent those of their affiliated organizations, or those of the publisher, the editors and the reviewers. Any product that may be evaluated in this article, or claim that may be made by its manufacturer, is not guaranteed or endorsed by the publisher.
